# Supplementary material for: SARS-CoV-2 mRNA Vaccine Response in People Living with HIV According to CD4 Count and CD4/CD8 Ratio
Source: Vaccines (Basel). 2023 Oct 30;11(11):1664. doi: 10.3390/vaccines11111664 (PMC10675416; doi:10.3390/vaccines11111664)
Supplement: Supplementary file 1 [file vaccines-11-01664-s001.zip › vaccines-2628925-supplementary.pdf]

Supplementary Table S1. Conversion factors for the different assays used

Company	Platform	Assay	Antigen	Assay Unit	Conversion factor to BAU/mL
**Abbott**	**Alinity /Architect**	SARS-CoV-2 (IgG)	Nucleocapsid	S/CO	Not available
		SARS-CoV-2 (IgG) QUANT II	RBD	AU/mL	0.142
**Roche**	**Elecsys**	Anti-SARS-CoV-2	Nucleocasid	U/mL	Not available
		Anti-SARS-CoV-2 S	RBD		1.0288
**MSD**	**V-Plex**	SARS-CoV-2 (Spike) (IgG)	Spike	AU/mL	0.00901
		SARS-CoV-2 (RBD) (IgG)	RBD		0.0272
		SARS-CoV-2 (N) (IgG)	Nucleocapsid		0.00236
**Diasorin**	**Liaison**	SARS-CoV-2 TrimericS (IgG)	Spike	BAU/mL	Not required

Supplementary Table S2. Odds Ratio (OR) and Adjusted Odds Ratio (AOR) from fitting a logistic regression model of the probability of failing to achieve a robust/above-average anti-S/RBD response post vaccination VE90% (**Panel A**) and OCOnAbs (**Panel B**) endpoints after excluding subjects and data quantified with Elecsys Anti-SARS-CoV2 S (Roche)

**Panel A (VE90%)**	**Unadjsuted Odds ratio (95% CI)**	**p-value**	**Adjusted* Odds ratio (95% CI)**	**p-value**	**&Type III p-value**
**CD4 count at T1**	***Fail to achieve VE90% after primary cycle (T3)***				
HCD4	1		1		0.019
ICD4	1.95 (1.43, 2.66)	<.001	1.58 (0.90, 2.76)	0.112	
LCD4	4.50 (2.37, 8.53)	<.001	3.93 (1.37, 11.26)	**0.011**	
per 1 SD lower (log2 scale)	1.72 (1.46, 2.03)	<.001	1.76 (1.27, 2.44)	**<.001**	
	Fail to achieve VE90% after first booster dose (T5)				
HCD4	1		1		0.937
ICD4	1.37 (0.89, 2.13)	0.157	0.91 (0.41, 2.03)	0.814	
LCD4	2.00 (1.08, 3.72)	0.028	0.81 (0.24, 2.75)	0.733	
per 1 SD lower (log2 scale)	1.42 (1.16, 1.74)	<.001	1.19 (0.78, 1.83)	0.421	
CD4 count at T1					

**Panel B (OCOnAbs)**	**Unadjsuted Odds ratio (95% CI)**	**p-value**	**Adjusted* Odds ratio (95% CI)**	**p-value**	**&Type III p-value**
**CD4 count at T1**	***Fail to achieve OCOnAbs after primary cycle (T3)***				
HCD4	1		1		0.403
ICD4	1.67 (1.18, 2.37)	0.004	1.48 (0.81, 2.68)	0.202	
LCD4	3.08 (1.50, 6.33)	0.002	1.48 (0.52, 4.23)	0.468	
per 1 SD lower (log2 scale)	1.47 (1.23, 1.76)	<.001	1.38 (0.99, 1.93)	0.057	
	Fail to achieve OCOnAbs after first booster dose (T5)				
HCD4	1		1		0.925
ICD4	1.29 (0.84, 1.98)	0.244	0.87 (0.40, 1.89)	0.730	
LCD4	1.72 (0.93, 3.19)	0.083	0.83 (0.26, 2.67)	0.759	
per 1 SD lower (log2 scale)	1.35 (1.11, 1.65)	0.003	1.19 (0.79, 1.80)	0.409	

**adjusted for age, VL<=50 copies/mL at T1,no. of comorbidities and nadir CD4 count; &from the adjusted model*

Supplementary Table S3. OR and AOR from fitting a logistic regression model of the probability of failing to achieve a robust/above-average anti-S/RBD response post vaccination VE90% (**Panel A**) and OCOnAbs (**Panel B**) endpoints after excluding subjects and data quantified with Elecsys Anti-SARS-CoV2 S (Roche)

Panel A (VE90%)	Unadjsuted Odds ratio (95% CI)	p-value	Adjusted* Odds ratio (95% CI)	p-value	&Type III p-value
**CD4/CD8 ratio at T1**	***Fail to achieve VE90% after primary cycle (T3)***				
HR	1		1		0.246
IR	1.21 (0.87, 1.66)	0.253	0.75 (0.42, 1.35)	0.346	
LR	1.72 (1.25, 2.36)	<.001	1.22 (0.66, 2.27)	0.526	
per 1 SD lower (log2 scale)	1.32 (1.16, 1.50)	<.001	1.18 (0.92, 1.53)	0.199	
	Fail to achieve VE90% after first booster dose (T5)				
HR	1		1		0.474
IR	1.12 (0.67, 1.89)	0.659	0.58 (0.23, 1.46)	0.247	
LR	1.50 (0.92, 2.45)	0.106	0.82 (0.33, 2.05)	0.677	
per 1 SD lower (log2 scale)	1.31 (1.09, 1.57)	0.004	0.98 (0.68, 1.41)	0.921	

Panel B (OCOnAbs)	Unadjsuted Odds ratio (95% CI)	p-value	Adjusted* Odds ratio (95% CI)	p-value	&Type III p-value
**CD4/CD8 ratio at T1**	***Fail to achieve OCOnAbs after primary cycle (T3)***				
HR	1		1		0.834
IR	0.99 (0.69, 1.41)	0.941	0.89 (0.49, 1.61)	0.699	
LR	1.26 (0.89, 1.80)	0.193	1.06 (0.56, 2.02)	0.853	
per 1 SD lower (log2 scale)	1.18 (1.02, 1.36)	0.024	1.02 (0.78, 1.34)	0.878	
	Fail to achieve OCOnAbs after first booster dose (T5)				
HR	1		1		0.396
IR	0.97 (0.59, 1.60)	0.900	0.65 (0.27, 1.56)	0.332	
LR	1.35 (0.84, 2.16)	0.216	1.06 (0.44, 2.55)	0.898	
per 1 SD lower (log2 scale)	1.27 (1.06, 1.52)	0.009	1.09 (0.77, 1.54)	0.635	

**adjusted for age, VL<=50 copies/mL at T1, no. of comorbidities and nadir CD4 count; &from the adjusted model*

Supplementary Table S4. Adjusted mean anti-RBD values from fitting a mixed-linear model EXCLUDING subjects and data quantified with Elecsys Anti-SARS-CoV2 S (Roche)

anti-RBD, log2	T1 95% CI	T2 95% CI	T3 95% CI	T4 95% CI	T5 95% CI	T6 95% CI	p-value*
CD4 at T1							**<.001**
LCD4	-4.8 (-5.7, -4.0)	1.0 (0.2, 1.8)	4.8 (4.0, 5.6)	2.4 (1.6, 3.3)	8.0 (7.1, 8.9)	5.6 (4.2, 7.0)	
ICD4	-5.2 (-5.7, -4.7)	3.2 (2.7, 3.7)	9.1 (8.5, 9.6)	7.0 (6.6, 7.5)	8.2 (7.3, 9.0)	4.4 (3.9, 5.0)	
HCD4	-4.8 (-5.2, -4.4)	4.3 (3.9, 4.7)	9.1 (8.6, 9.6)	7.4 (7.0, 7.7)	7.7 (6.8, 8.6)	4.7 (4.3, 5.2)	
CD4/CD8 ratio at T1							**0.012**
HR	-4.9 (-5.4, -4.5)	2.8 (2.4, 3.2)	6.5 (6.1, 6.9)	3.7 (3.3, 4.2)	8.7 (8.2, 9.2)	7.3 (6.5, 8.0)	
IR	-5.0 (-5.6, -4.4)	4.0 (3.4, 4.5)	9.2 (8.5, 9.8)	7.2 (6.7, 7.7)	8.0 (6.8, 9.3)	4.7 (4.1, 5.3)	
LR	-4.9 (-5.5, -4.3)	4.4 (3.8, 4.9)	9.3 (8.5, 10.0)	7.4 (6.9, 7.8)	7.7 (6.4, 9.0)	5.1 (4.4, 5.7)	

**F-test type 3 interaction p-value*

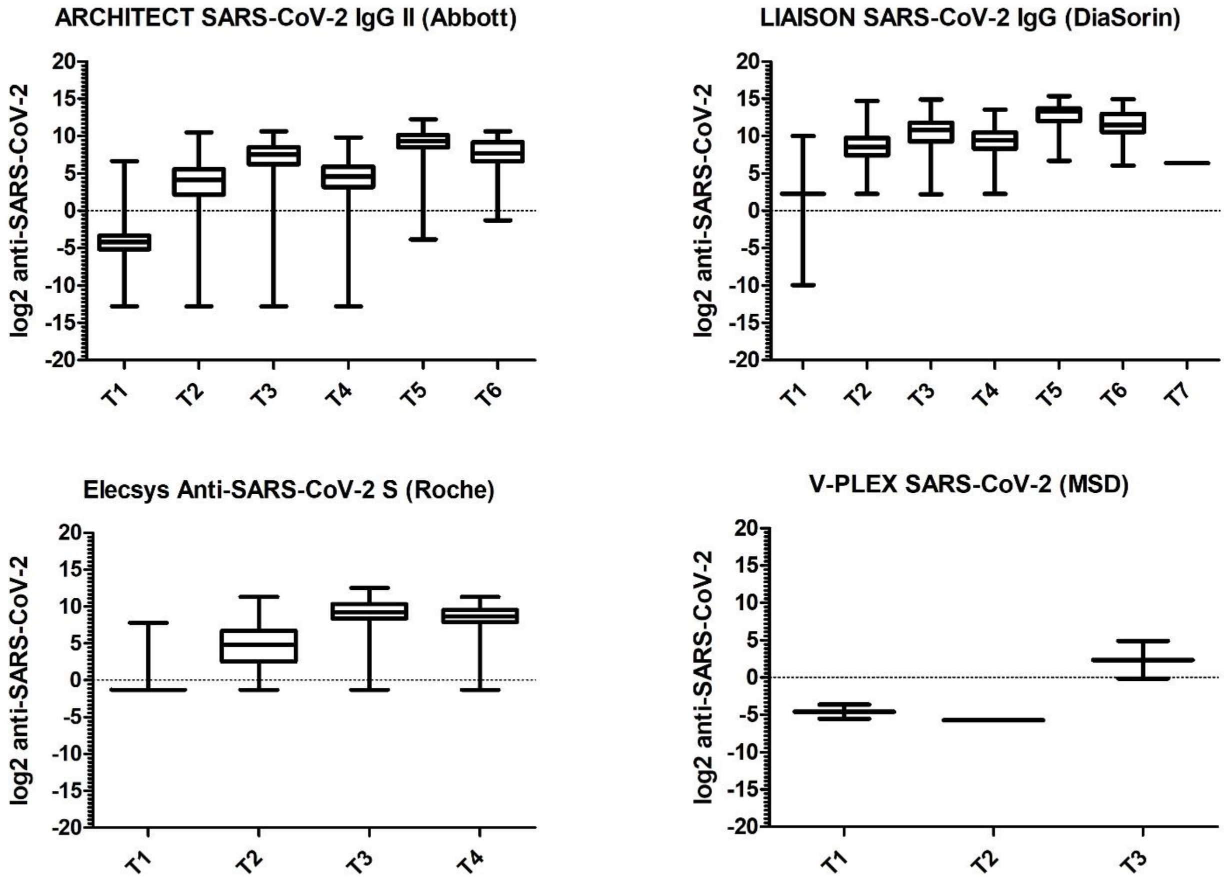

Supplementary Figure S1. Boxplots of Anti-SARS-CoV-2 titers (log2 transformation) according to the assay used

Supplementary Table S5. Median (IQR) quantification of anti-SARS-CoV-2 titers (log2 transformation) at different time points after vaccination according to immunoassay used and CD4 strata **(A)** and CD4/CD8 strata **(B)**

	Architect anti-SARS-CoV-2 IgG II (Abbott), anti-RBD IgG, log2, median (IQR)					
**(A)**	**T1**	**T2**	**T3**	**T4**	**T5**	**T6**
<u>**CD4 at T1**</u>						
**HCD4**	-4.14 (-5.14, -3.33)	4.61 (3.3, 5.76)	7.73 (6.59, 8.65)	4.86 (3.76, 6.01)	9.35 (8.55, 9.89)	7.71 (6.88, 8.89)
**ICD4**	-4.55 (-5.14, -3.55)	3.92 (1.51, 5.3)	7.35 (6.21, 8.52)	4.54 (3.02, 6.15)	9.56 (8.74, 10.48)	8.44 (7.11, 9.6)
**LCD4**	-3.82 (-4.55, -3.33)	1.59 (-0.97, 3.61)	6.17 (3.83, 7.46)	3.19 (1.81, 4.9)	9.27 (7.64, 10.03)	5.68 (5.36, 7.36)
	Liaison SARS-CoV-2 IgG (Diasorin), anti-S IgG , log2, median (IQR) anti-S IgG					
	**T1**	**T2**	**T3**	**T4**	**T5**	**T6**
**HCD4**	2.26 (2.26, 2.26)	8.77 (7.73, 9.76)	10.88 (9.43, 11.8)	9.44 (8.33, 10.28)	13.29 (12.02, 13.7)	11.29 (10.39, 13.21)
**ICD4**	2.26 (2.26, 2.26)	7.98 (6.79, 9.64)	10.75 (8.71, 11.79)	9.73 (8.61, 10.74)	13.04 (11.31, 13.68)	11.54 (10.87, 12.78)
**LCD4**	2.26 (2.26, 2.98)	6.4 (3.52, 7.82)	9.19 (5.17, 11.71)	9.56 (6.48, 10.77)	13.27 (12.33, 14.33)	11.82 (10.56, 12.97)

	Architect anti-SARS-CoV-2 IgG II (Abbott), anti-RBD IgG, log2, median (IQR)					
<u>**(B)**</u>	**T1**	**T2**	**T3**	**T4**	**T5**	**T6**
<u>**CD4/CD8 ratio at T1**</u>						
**HR**	-4.35 (-5.14, -3.33)	4.64 (3.49, 5.83)	7.7 (6.62, 8.56)	4.97 (3.82, 6.02)	9.35 (8.59, 9.89)	7.59 (6.88, 8.77)
**IR**	-4.35 (-5.14, -3.55)	4.62 (2.95, 5.73)	7.42 (6.16, 8.64)	4.82 (3.72, 6.00)	9.35 (8.62, 9.95)	7.95 (7.11, 9.76)
**LR**	-4.14 (-5.14, -3.33)	3.41 (1.01, 5.1)	7.32 (5.81, 8.43)	4.37 (2.79, 5.56)	9.52 (8.24, 10.26)	7.66 (6.05, 9.18)
	Liaison SARS-CoV-2 IgG (Diasorin), anti-S igG, log2, median (IQR)					
	**T1**	**T2**	**T3**	**T4**	**T5**	**T6**
**HR**	2.26 (2.26, 2.26)	8.81 (8.04, 9.72)	10.72 (9.13, 11.63)	9.44 (8.27, 10.2)	13.36 (11.41, 13.61)	11.22 (10.12, 12.92)
**IR**	2.26 (2.26, 2.26)	8.51 (7.42, 9.67)	10.88 (9.46, 11.79)	9.62 (8.61, 10.34)	13.08 (12.35, 14)	11.7 (10.56, 13.44)
**LR**	2.26 (2.26, 2.26)	8.04 (6.48, 9.76)	10.87 (9.36, 12.17)	9.54 (7.98, 10.74)	13.08 (11.46, 13.85)	11.54 (10.79, 12.78)
